# Vaccine effectiveness against severe COVID-19 during the Omicron wave in Germany: results from the COViK study

**DOI:** 10.1007/s15010-023-02012-z

**Published:** 2023-03-13

**Authors:** Anna Stoliaroff-Pepin, Caroline Peine, Tim Herath, Johannes Lachmann, Wiebke Hellenbrand, Delphine Perriat, Achim Dörre, Andreas Nitsche, Janine Michel, Marica Grossegesse, Natalie Hofmann, Thomas Rinner, Claudia Kohl, Annika Brinkmann, Tanja Meyer, Daniel Stern, Fridolin Treindl, Brigitte G. Dorner, Sascha Hein, Laura Werel, Eberhard Hildt, Sven Gläser, Helmut Schühlen, Caroline Isner, Alexander Peric, Ammar Ghouzi, Annette Reichardt, Matthias Janneck, Guntram Lock, Dominik Huster, Thomas Grünewald, Lars Schaade, Ole Wichmann, Thomas Harder

**Affiliations:** 1 Department for Infectious Disease Epidemiology, Immunization Unit, Robert Koch Institute, Berlin, Germany; 2 Department for Infectious Disease Epidemiology, Robert Koch Institute, Berlin, Germany; 3 Centre for Biological Threats and Special Pathogens, WHO Reference Laboratory for SARS-CoV-2 and WHO Collaborating Centre for Emerging Infections and Biological Threats, ZBS1 Highly Pathogenic Viruses, Robert Koch Institute, Berlin, Germany; 4 Centre for Biological Threats and Special Pathogens, ZBS3 Biological Toxins, Robert Koch Institute, Berlin, Germany; 5 Division Virology, Paul-Ehrlich-Institute, Paul-Ehrlich-Straße 51-59, 63225 Langen, Germany; 6 Klinik für Innere Medizin-Pneumologie und Infektiologie, Vivantes Klinikum Neukölln und Spandau, Berlin, Germany; 7 Direktorat Klinische Forschung & Akademische Lehre, Vivantes Netzwerk für Gesundheit GmbH, Berlin, Germany; 8 Klinik für Innere Medizin-Infektiologie, Vivantes Auguste-Viktoria-Klinikum, Rubensstr. 125, 12157 Berlin, Germany; 9 Klinik für Pneumologie und Infektiologie, Vivantes Klinikum im Friedrichshain, Landsberger Allee 49, 10249 Berlin, Germany; 10 Schön Klinik Düsseldorf, Interdisziplinäre Notaufnahme, Am Heerdter Krankenhaus 2, 40549 Düsseldorf, Germany; 11 Helios Klinikum Berlin-Buch, Schwanebecker Chaussee 50, 13125 Berlin, Germany; 12 Sektion Nephrologie, Klinik für Kardiologie, Albertinen Krankenhaus, Süntelstraße 11a, 22457 Hamburg, Germany; 13 Klinik für Innere Medizin, Albertinen Krankenhaus, Süntelstraße 11a, 22457 Hamburg, Germany; 14 Klinik für Innere Medizin, Helios Klinikum Erfurt, Erfurt, Germany; 15 Klinik für Infektions-und Tropenmedizin, Klinikum Chemnitz, Chemnitz, Germany; 16 Centre for Biological Threats and Special Pathogens, Robert Koch Institute, Berlin, Germany

**Keywords:** COVID-19, Vaccine effectiveness, Hospitalization, Case-control study, SARS-CoV-2, Omicron

## Abstract

**Purpose:**

COViK, a prospective hospital-based multicenter case-control study in Germany, aims to assess the effectiveness of COVID-19 vaccines against severe disease. Here, we report vaccine effectiveness (VE) against COVID-19-caused hospitalization and intensive care treatment during the Omicron wave.

**Methods:**

We analyzed data from 276 cases with COVID-19 and 494 control patients recruited in 13 hospitals from 1 December 2021 to 5 September 2022. We calculated crude and confounder-adjusted VE estimates.

**Results:**

21% of cases (57/276) were not vaccinated, compared to 5% of controls (26/494; *p* < 0.001). Confounder-adjusted VE against COVID-19-caused hospitalization was 55.4% (95% CI: 12–78%), 81.5% (95% CI: 68–90%) and 95.6% (95%CI: 88–99%) after two, three and four vaccine doses, respectively. VE against hospitalization due to COVID-19 remained stable up to one year after three vaccine doses.

**Conclusion:**

Three vaccine doses remained highly effective in preventing severe disease and this protection was sustained; a fourth dose further increased protection.

**Supplementary Information:**

The online version contains supplementary material available at 10.1007/s15010-023-02012-z.

## Background

The SARS-CoV-2 pandemic spread globally since December 2019 with more than 600 million cumulative cases and 6.8 million deaths documented by the end of January 2023 [1]. Several different variants of the virus evolved over time. In Germany, the Alpha and Delta variants dominated in 2021. In November 2021, the WHO declared lineage B.1.1.529, also called Omicron variant, as a variant of concern (VOC). From 12 December 2021 to 30 January 2022, the proportion of the Omicron variant in Germany increased from 2.3% to 97.8% and throughout the year 2022, Omicron was the dominant variant in Germany [2].

The Omicron variant differs from former variants by more than 30 amino acid modifications in the spike protein. It is characterized by many concerning epidemiological features like lower minimal infection dose resulting in higher transmissibility, immune evasion with the risk of reinfections and breakthrough infections, and an impaired response to COVID-19-specific treatment [3–5]. In contrast to other variants, virus replication of the Omicron variant occurs mainly in the upper respiratory tract (e.g. pharynx), which may lead to higher transmission rates and milder disease [5–8].

Vaccine effectiveness (VE) against the wild-type virus and the Alpha, Beta and Delta variants was very high irrespective of outcome definition [9, 10]. Previous studies showed decreased VE against the Omicron variant due to mutations in the spike protein, but effectiveness against severe disease remained high [11, 12].

More detailed, individual data are needed to allow valid conclusions about the VE in subgroups with proper statistical adjustment for confounders. We therefore launched a case-control study in 13 hospitals across Germany (COViK) to assess the effectiveness of vaccines in preventing COVID-19-associated hospitalization in the adult population. In addition, we performed subgroup analyses and studied different time intervals of protection. Initial data collected during the Delta wave were published recently [9]. Here, we present the profile of hospitalized COVID-19-patients and COVID-19 VE based on data obtained during the Omicron wave.

## Methods

### Study design

We conducted a prospectively recruiting multi-center case-control study with 1:2 matching in 13 hospitals in five federal states (Berlin, Hamburg, North Rhine-Westphalia, Thuringia, Saxony) in Germany. For this interim analysis, we used data of patients recruited during the Omicron wave.

Study nurses were trained by the COViK study center team at the beginning of the assignment. Additionally, on-site audits were conducted regularly and study nurses underwent several in-house trainings.

Inclusion/exclusion criteria and matching: patients were eligible to be included as cases, if they were aged between 18 and 90 years and hospitalized due to laboratory confirmed, symptomatic SARS-CoV-2 infection (COVID-19) at one of the 13 study hospitals from 1 December 2021 to 5 September 2022 (for details, see Suppl. Methods). Directly after the inclusion of a case, two SARS-CoV-2 negative patients in the same hospital as the case (or a hospital in the same city) were recruited as controls. These controls were further matched by age, sex and admission date (for details, see Supplement).

### Biological samples and data collection

Nasopharyngeal and oropharyngeal swabs were taken from cases and controls. SARS-CoV-2 PCR and sequencing were performed at the Robert Koch Institute [13, 14] (for details, see supplement). Information about socio-demographic factors, vaccination history, risk factors for COVID-19 infection and severe course of disease were collected during interviews. Clinical and laboratory data were extracted from medical records. The Charlson comorbidity index was used to represent possible comorbidities in our questionnaire [15].

Study nurses validated the information on vaccination with the vaccination certificate or vaccination app (CovPass App or Corona-Warn-App, both provided by the German government).

### Statistical analysis

Comparisons between cases and controls were performed using appropriate significance tests (unpaired Student’s t-tests for age and BMI and Chi-squared test or Fisher's exact test for age group, sex, educational level, number of comorbidities, vaccination status and number of vaccine doses).

We computed the 2-dose, 3-dose, 4-dose and 2 vs. 3 or 4 dose-VE for all patients, as well as for the following subgroups: males/females; age 18-59/60-69/70-90 years; <3/ ≥3 pre-existing comorbidities; admission to intensive care (ICU)/non-ICU ward. For assessment of the duration of vaccine-induced protection, VE was estimated according to symptom onset 14–90 days, 91–180 days and 181–365 days after the last vaccine dose. Patients who received only one vaccine dose were included in the descriptive analysis but excluded from VE analysis.

As the matched subgroup-analysis was not applicable for all subgroups due to insufficient number of patients in the strata, we primarily performed an unmatched analysis as also suggested by others [16]. The odds ratio (OR) to prevent severe COVID-19 by vaccination was calculated with the formula:

$$OR= \frac{\frac{vaccinated \,cases}{unvaccinated \,cases}}{\frac{vaccinated \,controls}{unvaccinated \,controls}}$$

The VE was subsequently calculated with the formula

$$VE \, = \, \left( {1 - OR} \right) \times 100$$

To assess the robustness of results, pairwise matched analysis was conducted (see Supplement Tables 3 and 4, Figure 4) using the Mantel–Haenszel method:

Matched pairs odds ratio: $\frac{b}{c}$
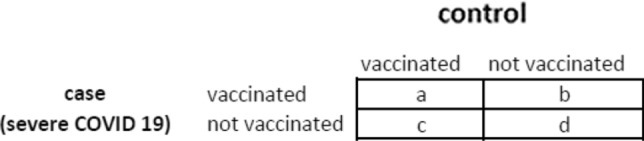


In some subgroup analyses, we applied the Woolf–Haldane correction as the combination vaccinated case(s)/unvaccinated control(s) was not present in every subset of the data.

Immunocompromising comorbidities/therapies and diseases without immunosuppression were analyzed separately. To determine the minimal set of relevant confounder variables for the adjustment, we constructed a directed acyclic graph (DAG; see Supplement Figure 2). Accordingly, estimation of VE was adjusted for age group, socio-economic status (education), pre-existing comorbidities and risk of infection (surrogate parameter, e. g. profession, daily high-risk activities without masking, housing situation; see also Suppl. Methods). For four variables, adjustment was already performed through matching (sex, region, phase of pandemic, infection protection recommendations).

Data were analyzed using the statistical software *R*, version 4.1.2.

## Results

**Fig. 1:**
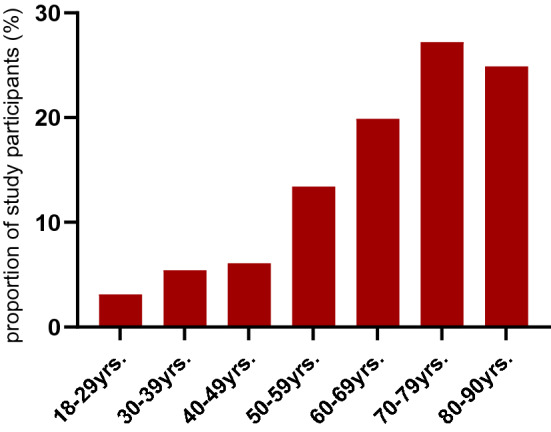
Age group proportions in the Omicron wave (cases)

We recruited 770 participants, comprising 276 cases and 494 matched controls (Table 1) during the specified study period. The median age of cases was 71 years and of controls 68 years (Fig. 1).

**Table 1:** Characteristics of cases and controls

	Cases	Controls	*p*-value
*n*	276	494	
Age (years) mean (SD)	66.65 (16.18)	65.25 (15.28)	0.233
Age group *n* (%)			
18–59 years	78 (28.3)	150 (30.4)	0.355
60–69 years	55 (19.9)	114 (23.1)	
70–90 years	143 (51.8)	230 (46.6)	
Sex *n* (%)			
Male	155 (56.2)	291 (58.9)	0.506
Female	121 (43.8)	203 (41.1)	
Highest school educational level *n* (%)			
No graduation	16 (5.8)	16 (3.2)	
9 school years	69 (25.0)	113 (22.9)	0.092
10 school years (secondary school certificate)	128 (46.4)	218 (44.1)	
12 or 13 school years (high school graduation)	63 (22.8)	147 (29.8)	
BMI (kg/m^2^) mean (SD))	26.93 (6.14)	29.00 (18.11)	0.066
BMI group *n* (%)			
Underweight/normal weight (BMI ≤ 25 kg/m^2^)	124 (44.9)	188 (38.1)	0.131
Overweight (BMI > 25-30 kg/m^2^)	79 (28.6)	171 (34.6)	
Obese (BMI > 30 kg/m^2^)	73 (26.4)	135 (27.3)	
Admission to intensive care unit (ICU) *n* (%)			
Yes	23 (8.3)	NA	
No	253 (91.7)	NA	
Death *n* (%)			
Yes	9 (3.3)	NA	
No	267 (96.7)	NA	
Pre-existing comorbidities (general) *n* (%)			
< 3	166 (60.1)	350 (70.9)	0.003
≥ 3	110 (39.9)	144 (29.1)	
Pre-existing comorbidities (immune system) *n* (%)			
None	191 (69.2)	414 (83.8)	< 0.001
≥ 1	85 (30.8)	80 (16.2)	
Vaccination (≥ 2 doses) *n* (%)			
Yes	206 (74.6)	461 (93.3)	
No	70 (25.4)	33 (6.7)	<0.001
Number of vaccine doses *n* (%)			
0	57 (20.7)	26 (5.3)	< 0.001
1	13 (4.7)	7 (1.4)	
2	46 (16.7)	49 (9.9)	
3	145 (52.5)	350 (70.9)	
4	15 (5.4)	62 (12.6)	
Vaccine type *n* (%)			
One dose (mRNA or vector)	13 (4.7)	7 (1.4)	
Two doses (mRNA/mRNA)	42 (15.2)	41 (8.2)	
Two doses (vector/vector)	1 (0.3)	4 (0.8)	
Two doses (crossover^b^)	2 (0.7)	4 (0.8)	
Three doses (mRNA/mRNA/mRNA)	126 (45.6)	281 (56.8)	
Three doses (mRNA/mRNA/vector)	0	1 (0.0)	
Three doses (vector/vector/mRNA)	10 (3.6)	29 (5.8)	
Three doses (crossover^b^/vector)	1 (0.3)	0 (0)	
Three doses (crossover^b^/mRNA)	7 (2.5)	39 (7.8)	
Not vaccinated	57 (20.6)	26 (5.2)	
Four doses, other vaccine or missing information	17 (6.1)	62 (12.5)	

^a^High-risk comorbidities for severe course of COVID-19: diabetes type 2, Organ transplant, BMI > 30, COPD, renal insufficiency, heart failure[17]

^b^first dose mRNA, second dose vector vaccine or vice versa

Of cases, 44% were female and of controls, 41%. Cases and controls were similar regarding their level of education, with 31% of cases and 26% of controls reporting nine or fewer school years (*p* = 0.092, Table 1).

Regarding prior SARS-CoV-2 infections, the rate was higher in controls (97/494 (19.6%) than in cases (13/276; 4.7%, Table 2). Seven participants were not able to report the date of prior infection.

**Table 2:** Prior infections and in temporal context with vaccination

	No prior infection		Unvaccinated with prior infection(s)		Prior infection date(s) before the first vaccination		Prior infection date(s) after the last vaccination		Prior infection between vaccinations	
	Cases	Controls	Cases	Controls	Cases	Controls	Cases	Controls	Cases	Controls
No vaccination	53	18	3	8	–	–	–	–	–	–
One vaccine dose	10	4	–	–	2	2	1	0	–	–
2 vaccine doses	44	32	–	–	1	8	1	7	0	1
3 or 4 vaccine doses	155	337	–	–	1	5	4	52	0	14
sum	262	391	3	8	4	15	6	59	0	15

**Fig. 2:**
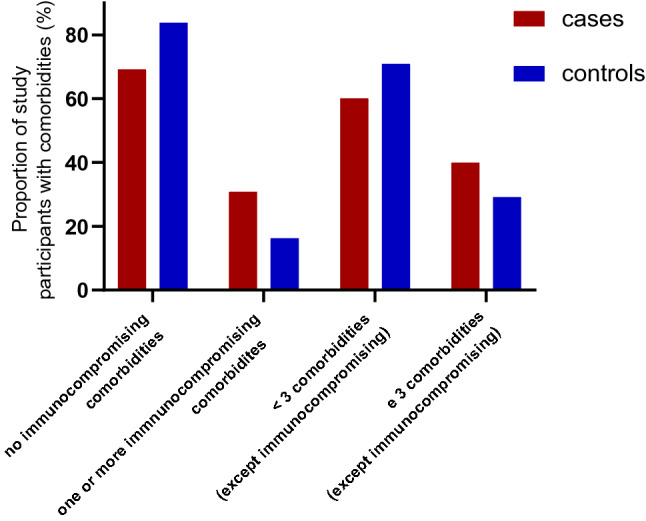
Comorbidities of study participants in the Omicron wave, cases vs. controls

Immunocompromising comorbidities were more prevalent in cases (31%) than in controls (16%, *p* < 0.001, Fig. 2, Table 1). Other comorbidities (e. g. heart failure, renal insufficiency, diabetes mellitus) were also more frequent in cases compared to controls (cases with ≥ 3 comorbidities 40%, controls 29%, *p* = 0.003, Fig. 2, Table 1).

Intensive Care Unit (ICU) treatment was necessary for 23 cases (8.3%). Nine cases died during their hospital stay (3.3%, Table 1).

Subtyping showed most cases to be infected with Omicron belonging to the BA.1 and BA.2 subgroups (Supplement Figure 3).

**Fig. 3:**
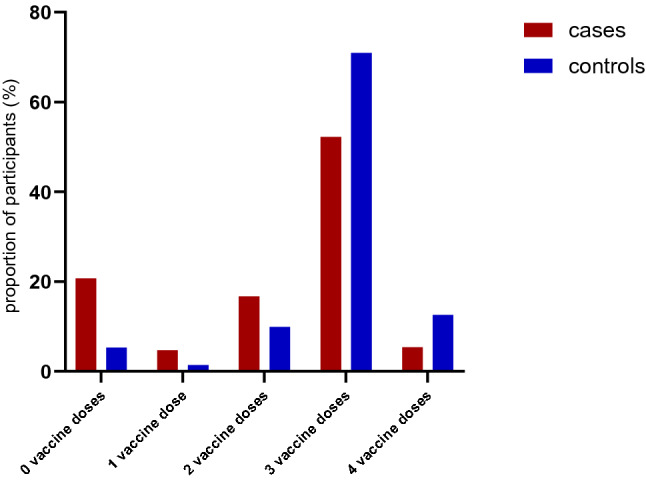
Number of vaccine doses in cases (*n* = 276) and controls (*n* = 494)

Vaccination status differed significantly between cases and controls: 21% of the cases were not vaccinated at all, compared to 5% of the controls (*p* < 0.001, Figure 3). Moreover, only 53% of cases, compared to 71% of controls, had received three doses of vaccine. 5% of cases and 13% of controls had received four vaccine doses (Figure 3). The difference between the proportions of boostered (three or four doses) cases and controls was statistically significant (*p* < 0.001).

For 238 participants (35%), information on vaccination was verified by the vaccination certificate. In 374 participants (54%), the vaccination app was used for verification, and in 14 participants (2 %), this information came from the general practitioner. It was not possible to verify the data of 61 vaccinated participants (9%). 83 participants were not vaccinated.

**Fig. 4:**
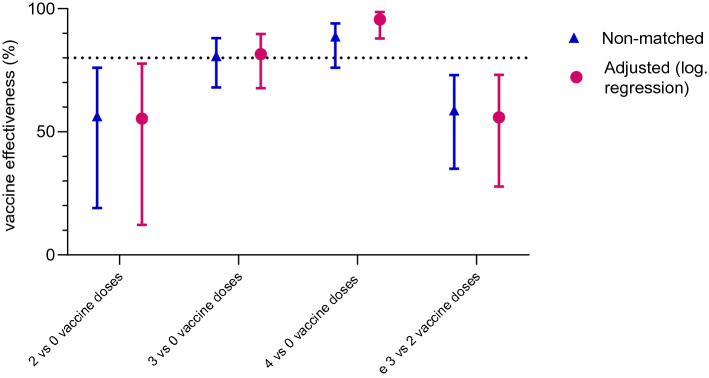
Vaccine effectiveness: unmatched analysis, crude versus confounder adjusted logistic regression

In the unmatched crude analysis, two-dose VE against severe disease was 56.4% (95% CI: 19–76%) three-dose VE was 80.8% (95% CI: 68–88%) and 4-dose VE was 88.7% (95% CI: 76–94%). Compared to 2 doses, VE was 58% (95% CI: 35–73%) for ≥ 3 doses (Fig. 4). For further details, see Supplement Tables 1 and 2).

After adjustment for confounders, overall VE in the Omicron wave was similar to the results of the crude analysis (Fig. 4). The adjusted VE against severe disease was 55.4% (95% CI: 12–78%) after two, 81.5% (95% CI: 68–90%) after three and 95.6% (95% CI: 88–99%) after four doses compared to non-vaccinated.

The results of the pairwise-matched analysis were similar to those of the unmatched analysis (see Supplement Tables 3 and 4, Supplement Figure 4).

VE after four doses was about 90% in all subgroups, with the highest estimate in ICU-treated patients (96.5%; 95% CI: 35–99%), and in the age group > 70 years (94.9%, 95% CI: 85–98%). Four-dose VE estimates were higher (although not significantly) than 3-dose VE in all subgroups (Supplement Table 2).

**Fig. 5:**
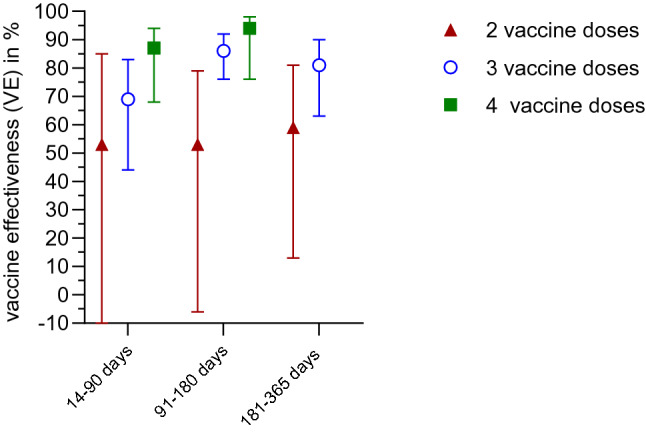
Time between last vaccine dose and symptom onset: vaccine effectiveness for different time intervals

When VE estimates were stratified according to time interval between last vaccine dose and date of symptom onset, we observed no significant differences between VE values, indicating a stable VE over time for up to one year. As shown in Figure 5, this was observed for two and three vaccine doses. Six to twelve months after the last vaccine dose, VE was 59.6% (95% CI: 13–81%) for two doses and 81% for three vaccine doses (95% CI: 63–90%, Supplement Tables 1 and 2).

## Discussion

To our knowledge, this is the first study in Germany to assess the VE of COVID-19 vaccines against severe disease caused by the Omicron variant in the post-marketing phase. Our analysis suggests that three doses of COVID-19 vaccines are highly effective in preventing hospitalization due to COVID-19 and that protection remains stable for up to one year. A fourth dose further increases the protective effect.

The proportion of older patients and cases with immunocompromising comorbidities in the Omicron wave was higher compared with our data from an earlier recruitment phase in the Delta wave [9]. This difference in clinical characteristics was confirmed in other studies [18, 19]. In settings with a high immunization coverage and a highly transmissible variant such as Omicron, immune-compromised and older persons were more often affected by severe COVID-19. Patients with comorbidities often have a fragile health situation and even a mild or moderate SARS-CoV-2 infection can impair the state of health and lead to hospital admission with COVID-19 as the main diagnosis. However, the lower pathogenicity of the Omicron variant and the advanced therapeutic possibilities still led to milder disease in the Omicron wave compared with the Delta wave [9, 18–20].

About 20% of controls and 5% of cases reported a prior SARS-CoV-2 infection. This imbalance is likely to be caused by the study design as well as high vaccine effectiveness. Compared to controls, cases were mostly unvaccinated or incompletely vaccinated and were in a more vulnerable health status. Therefore, cases had a higher probability to have a severe form of COVID-19, leading to hospitalization already when they were infected for the first time. In contrast, controls, by definition, did not have a severe infection leading to hospitalization. This might have been the result of both, higher vaccination coverage and prior infections. Although prior infections might confound the result in the controls, as the probability of being vaccinated is lower after infection with consequential underestimation of VE and immunity after infection can reduce the probability of (severe) infection (effect modifier), we decided against excluding participants with prior infection to avoid selection bias,. e.g. selection of more careful patients with fewer contacts, presumably persons with a high risk of severe course of disease. We decided against adjusting for prior infection, because the fact, that most of the cases experience severe first infections and only a part of the controls experienced a mostly mild infection is inherent in the study design and adjusting would bias the results. Additionally, the information about prior infections are anamnestic and likely to be incomplete (e.g. asymptomatic or mild infections).

The proportion of unvaccinated cases and controls was markedly lower in the Omicron wave compared to the Delta wave [9] and the percentage of boostered cases and controls increased. The increase might be related to the awareness campaign for vaccination in Germany and to increasing confidence in the vaccines. Nevertheless, as only 8% of the cases with an immunocompromising comorbidity had received a fourth vaccine dose, the vaccination coverage of patients at risk for severe COVID-19 can still be increased.

Whereas two vaccine doses reduced the likelihood of severe COVID-19 by 55% (95% CI: 12–78%), the effectiveness was higher for three (81%, 95% CI 68–90%) and especially four doses (97% after adjustment, 95% CI: 88–99%). In the analysis of our data from the Delta wave VE was higher for two doses (89%, 95% CI: 84–92%) and three doses (97%, 95% CI 95–99%). The results from the Omicron wave analyses were comparable with the results of other studies and systematic reviews [21–25].

A systematic review from Shao et al. including 113 studies found a lower pooled VE for Omicron (two doses 56% and three doses 83%) compared to the VE against the Delta variant (85% and 93%, respectively) for COVID-19 related hospitalization [24].

Recent studies using register data confirm that the 3-dose VE in the Omicron wave ranges between 80 % and 90 % [21, 22, 23]

When Omicron was predominant, VE estimates for three vaccine doses were around 80% in our study. However, effectiveness was restored by a fourth vaccine dose (VE 96%). It must be taken into account that the fourth dose was only administered to patients at risk (over 70 years or with comorbidities) in the recruitment phase, meaning that patients with four doses were a highly selected and rather small group.

Our analysis revealed stable protection up to one year for three vaccine doses. As other studies showed that protection against symptomatic infection decreases noticeably with increasing time lag after the last vaccination dose [22, 23, 25–27], this is an important finding.

Additionally, our subgroup analyses (different age groups, sex, with low and high comorbidity burden, with and without ICU treatment) reveled a comparably high protection for all subgroups with VE between 75 and 92% for three vaccine doses (Supplement Table 1).

One of the main advantages of our study design is the prospective collection of detailed high-quality patient data. This permitted flexible adaptation to the different pandemic phases when the circumstances made adaptation unavoidable. To ensure high data quality, each COVID-19 diagnosis was confirmed by clinical records and—where necessary—by direct consultation of the attending physician. Only patients requiring hospitalization due to COVID-19 were included. Many post-marketing studies rely on clinical data registries, allowing a fast analysis of large datasets. However, especially in the Omicron wave, register studies have limitations. Studies based on ICD-10 code assessment can be biased through misclassification, as the coding is designed to document billing. SARS-CoV-2 positive hospitalized patients with underlying comorbidities can be misclassified as severe COVID-19 cases, although the underlaying disease is in the foreground. Thereby, VE against severe COVID-19 is likely to be underestimated in these studies, as VE against mild COVID is known to be lower.

Our study is one among few to adjust for risk of infection. The minimal set of variables for adjustment was determined by constructing a DAG (directed acyclic graph). The variable “infection risk” was necessary to build the DAG and obtain a minimal sufficient adjustment set. However, a proper assessment is not trivial since factors which influence risk of infection change over time.

The comparison of three different methods of analysis showed the robustness of the results: the results of the pairwise-matched method were very similar to those of the non-matched analysis, but—as expected—not as precise [16]. For the pairwise-matched analysis, a large dataset is necessary to permit analysis of subgroups.

Our study has some limitations. The sample size is small, especially in the subgroups, so interpretations have to be done carefully and not every desired subgroup analysis is possible.

As selection biases are one of the major causes of bias in case–control studies, we explain our approach to reducing the risk of bias and especially considerations concerning selection bias in the following. Selection biases in case-control studies assessing VE occur especially in influenza studies with outpatients, as the patient characteristics (e. g. socio-economic status) influence patients in their decision to visit a doctor or not. For this reason, test-negative design studies are preferred in this setting as they include people with a comparable health-seeking behaviour. In contrast to this, the endpoint of our study (hospital admission) is less vulnerable towards a selection bias, as the decision of hospital admission is not strongly associated with socio-economic status and related characteristics. In addition, we expected a selected patient group (e.g. bedridden patients with endogenous pneumonia) of test-negative patients in the pandemic as most respiratory infections were prevented by non-pharmaceutical interventions. We therefore decided against a test-negative design.

Selection bias is also a risk when a specific group of eligible patients cannot be recruited into the study. In our case, patients who were not admitted to the hospital despite severe disease, patients with a fulminant course of disease or disoriented patients who could not sign the informed consent and did not have a legal representative could not be recruited. Patients who refuse vaccination often refuse to participate in studies, too. This might lead to a selection bias at a vaccine coverage greater than 85–90% [28, 29]. Furthermore, unvaccinated people in a population with high vaccination coverage may also have a different risk compared to the general population as these patients might not adhere to non-pharmaceutical interventions. We took countermeasures in distributing incentives to participants and offered training courses to study nurses to support participant recruitment.

In a case-control design, in contrast to test-negative studies, one should consider selection bias with regard to exposure to the virus. As the prevalence was high in the recruitment phase and we adjusted additionally for seven factors related to the risk of infection (e. g. profession, daily activity without mask, housing situation; details in the Supplement), we hypothesize a low risk of bias in this respect.

Previous SARS-CoV-2 infection was not an exclusion criterion as this would have led to a selection of patients who had presumably fewer contacts, e. g. high-risk patients. SARS-CoV-2 prevalence was high during recruitment and the exclusion of recovered controls may have led to overestimation of VE as recovered patients have a lower likelihood of vaccination. However, studies comparing controls with or without previous infection reported similar results [30].

To avoid patient selection bias by the study nurse in the hospital, the potential participants were recruited in a given order (day of the birth date). Every week, study nurses filled-in a non-responder list where reasons for non-participation were documented, irregularities were followed by site visits with quality control.

To avoid a selection of patients with a language barrier, an interpreter service with specially trained interpreters was employed that enabled the translation of conversations with patients in 40 languages. In addition, the questionnaires were translated into seven languages.

As controls were usually matched in the same hospital, bias due to regional differences could be avoided. Since elective patients might be more careful with personal contacts before the hospital admission, we instructed the study nurses to preferably recruit non-elective patients.

## Conclusion

High vaccination coverage, hybrid-immunity and evolution of viral variants changed the spectrum of patients with COVID-19 in the hospitals. Patients in the Omicron wave were significantly older and had more comorbidities compared to those in the Delta wave. Nevertheless, the course of disease was milder. COVID-19 vaccines were altogether highly protective against hospitalization in real-world settings in Germany during Omicron-variant predominance. High VE was also observed in subgroup analyses and remained stable for up to one year. The results were robust in the matched as well as in the unmatched analysis. However, the VE was lower compared to the Delta wave, which can be explained by partial immune evasion of the Omicron variant. In further analyses, we will investigate the impact of vaccination to prevent long COVID among our study participants.

## Supplementary Information

Below is the link to the electronic supplementary material.

Supplementary file1 (PDF 680 KB)

## Data Availability

Data will be made available on request.
